# The impact of the introduction of bilingual learning on sixth grade educational achievement levels

**DOI:** 10.1371/journal.pone.0234699

**Published:** 2020-06-16

**Authors:** María-Carmen García-Centeno, Laura de Pablos Escobar, Nuria Rueda-López, Carmen Calderón Patier

**Affiliations:** 1 Department of Applied Mathematics and Statistics, School of Business and Economy, CEU Universities, Madrid, Spain; 2 Department of Applied Economics Public and Political, Complutense University of Madrid, Madrid, Spain; 3 Department of Economics and Business, University of Almeria, Almeria, Spain; 4 Department of Economics, School of Business and Economy, CEU Universities, Madrid, Spain; Kyoto University, JAPAN

## Abstract

Bilingualism was implemented in the Community of Madrid (Spain) more than ten years ago, through the incorporation of the English language in the teaching methods of certain schools. Since that time, various research projects have been carried out, with the objective of comparing the academic performance of students in bilingual schools with those in non-bilingual schools. The present paper makes use of primary education data from the Department of Education and Research for the Community of Madrid in an effort to analyze whether or not bilingualism results in the relative improvement of educational outcomes achieved in primary schools in the Region of Madrid, in Spain. More specifically, the data used is from sixth grade classrooms, given that, generally speaking, in this grade all schools give a standardized test which measures academic performance in Math, Science and Technology, Spanish Language Arts and English Language Arts. Our assessment makes use of a multinomial logit model, and includes the most common variables found in the research on the determination of educational outcomes (variables related to whether or not schools are bilingual, which is the main focus of this paper), as well as other less common variables considered to be relevant. These include absenteeism, satisfaction levels among families and students, and the percentage of students in second chance programs. The results show that bilingualism does not lower children performance in the subjects taught in English or in the subjects taught in Spanish. Academic performance in Mathematics, Science and Technology, and Spanish Language Arts is similar with respect to those schools which are not bilingual. However, results in English are significantly higher in bilingual schools when compared to non-bilingual schools.

## Introduction

In recent decades, the application of bilingualism in various educational systems has become more common in many European countries, and most especially in Spain. Generally speaking, the method has been implemented through a program known as Content and Language Integrated Learning (CLIL), whose main objective is the simultaneous learning of a foreign language (usually English), along with the curriculum for each grade level. There are significant differences when it comes to how the program is implemented, depending on the social and educational context of the various participating countries [1].

In keeping with European trends, Spain incorporated CLIL into its educational system a couple of decades ago, although differing versions of the program can be found depending on the Spanish region, given that education is a de-centralized jurisdiction. For example, the number of subjects taught in English varies from region to region, as well as the level of foreign language that is expected not only from students, but from teachers [1]. In addition, there are peculiarities resulting from the fact that there are regions in Spain with two official languages. These include Catalonia, Valencia, the Balearic Islands, Galicia and the Basque Country [2]. Regions with a single official language include Andalusia, Madrid and Extremadura, among others [3].

The present research is rooted in the study of outcomes achieved in the bilingual system, specifically in terms of academic content and foreign language scores in a primary school setting in the Region of Madrid, Spain. The main characteristic of the program in primary education is that English is used to teach a minimum of 30% of the curriculum, preferably in the following subjects: Natural Science, Social Science, Physical Education, Art, Religion and Social and Civic Values. By contrast, Mathematics and Spanish Language Arts are taught in Spanish. In each school, the program begins in first grade, and is gradually expanded until sixth grade [4].

During the 2004–2005 academic year, the bilingual system was introduced in 26 public primary schools under the Order 796/2004 of the 5^th^ of March, by the Department of Education of the Region of Madrid. Since its implementation, the Department of Education has published a yearly list, detailing the order of new public schools to be integrated into the bilingual system. The list establishes the requirements to be met by each school to ensure the proper rollout of the program. During the 2008–2009 academic year, the program was extended to include charter schools. Later, in 2010–2011, Spanish-English bilingual education was offered to first year Compulsory Secondary Education (ESO) students in the public system in an effort to ensure continuity, and first year Baccalaureate students began to have access in 2014–2015. During the 2016–2017 academic year, the bilingual program also began to be introduced in Vocational Training courses, and in 2017–2018, 35 public primary schools started to offer Spanish-English education to children as young as four years old.

It is important to emphasize that bilingual programs that are part of the CLIL system, which is the case in the Region of Madrid, differ from other types of bilingual education like the immersion programs in Canada and the sort of bilingual education found in North America. While immersion programs impart the majority or all school subjects in the foreign language for a given length of time [5, 6], the CLIL program integrates language and content throughout a continuous, flexible process without an implicit preference for either of the two languages [7]. Other differences between the two methods include a later start time for CLIL and the fact that the materials used in this program are neither original nor taken from the daily life or culture of the target language, but are rather developed from an academic perspective [8].

In the context of this paper, our analysis uses the term bilingualism to refer to programs that are implemented in the Region of Madrid as part of CLIL, an approach whose characteristics have been defined previously. It is important to highlight, however, that when the literature is updated in the future, the term bilingualism will be used by other researchers to refer to specific programs with characteristics that may be different, depending on the geographical area in which they are implemented.

The objective of our research is to explore the factors that influence the probability of general improvement in academic outcomes and the learning of a foreign language in primary school, specifically in those schools which are included in the CLIL program in the Region of Madrid. The academic outcomes that have been used in this paper were obtained from assessments carried out in sixth grade classrooms in the Region of Madrid. These census tests are taken by all students, and include three sections: linguistic competence, mathematical competence, and basic science and technology competence.

The linguistic competence assessment, which includes both Spanish and the first foreign language (English), focuses on the two skills that delineate the field of communicative competence: comprehension and expression. As such, it assesses both oral and written comprehension skills.

The mathematical competence assessment includes the application of mathematical knowledge and reasoning for solving problems in functional contexts related to daily life. The student’s ability to carry out cognitive processes using their own knowledge will be assessed, as well as the reproduction of definitions, concepts and mathematical procedures.

The basic science and technology competence assessment includes the evaluation of abilities directed at the generation of scientific knowledge through gathering information, making hypotheses, problem solving and making decisions based on proof and debate.

All of the information laid out in the questionnaires for these tests, as well as the results, can be seen in detail on the following website: https://www.educa2.madrid.org/web/estadistica-ensenanza

We have considered that bilingualism could be one of several key factors, which we have divided into three groups: individual and educational factors (size of school, absenteeism, whether or not the student is repeating a grade, is an immigrant, or attended a nursery, etc.); socio-economic factors (parents’ educational level or quality of employment, etc.); and institutional variables (who the school is owned or run by, correction methods, etc.).

The structure of the present paper is as follows: After this introduction, in the second section, a review of existing empirical literature is carried out, highlighting the importance of bilingualism as a determining factor in educational outcomes. In the third section, statistically significant variables are determined for the academic performance of sixth grade students using a multiple regression model, and an analysis is carried out as to the effect of these outcomes on the probability of improvement using a multinomial logit model. Finally, the main results are laid out and discussed.

## Analysis of determining factors in academic performance. Recent history

The study of possible influential factors in scholastic performance (understood as achievements measured in numerical scores on relevant tests) has generated considerable interest in the academic world since the appearance of such pioneering studies as those by Coleman [9] and Hanushek [10]. The factors analyzed are extremely diverse, and take into account not only variables having to do with the circumstances surrounding both individuals and schools, but the character of each institution and the socio-economic environment of students. Bilingualism as it has been implemented in Spain, which is the main focus of this study, can be considered either as a school factor, or as an individual factor. Until now, this latter consideration has not been studied closely in Spain, but there is evidence of growing interest.

The literature cites the following individual and school factors as being the most common: the available means in schools to analyze the amount of public money spent on education [11], with an appreciation for the fact that differences in financial, personal and material resources as applied to education do not constitute a relevant factor in the explanation of educational outcomes, which is confirmed by various authors and the OCDE itself [12]. The availability of new technologies [13] has also been studied, with varying results [14, 15]. On an individual level, there is an interest in the influence of where students are from, whether from Spain or elsewhere. Most research points to lower performance from students who are immigrants [16, 17, 18, 19] and the gap only widens when the proportion of immigrants in a given school is greater than 20% [20, 21]. Some studies [22, 23], even highlight the fact that the effects are different depending on the student’s country of birth, although researchers are careful to point out it is individual and family variables that are best suited to explain outcomes [24, 25]. Other factors that have been studied recently regarding schools and individuals include representative levels of satisfaction with the good relationship between students and teachers, the implementation of orderly classes and clear explanations. All of these are important predictors when it comes to favorable outcomes [26]. This is equally true in the areas of sports and healthy eating habits for students [27, 28, 29, 30]. Rates of school dropout and students who repeat a grade are so considered significant variables that exert a negative influence on school performance [20, 31, 32].

Bilingualism in Spain can be considered as a variable related to the school or the individual that has not been analyzed in depth given its recent implementation, although several studies of note have already been carried out. In the region of Madrid, the influence of bilingual learning has been analyzed on the academic outcomes. These outcomes have been obtained on the Test for Essential Knowledge and Skills which is taken every year for student on sixth grade [33]. Students who started studying in the bilingual program in academic years 2004–2005 and 2005–2006, took the exam in 2009–2010 and 2010–2011, respectively. Conclusions indicate a negative effect in the outcomes of subjects taught in English for students whose parents have a lower level of education (less than a high school diploma). Nevertheless, no significant effects have been noted when it comes to performance in Reading and Mathematics, subjects which are taught in Spanish. These results are similar to analyses undertaken [34] in other regions of Spain including Asturias [35] and Extremadura [36], with performance levels that are slightly lower in bilingual schools for Science and Technology. Other studies show opposing results [37], with positive effects in the bilingual system both in Spanish Language Arts and in those subjects taught in English. The same results can be seen in secondary school education, with positive effects on certain non-cognitive skills among students: improvement in time spent enjoying reading and other positive habits related to this activity [38].

As such, results are not conclusive when it comes to the efficacy of bilingual programs, although the differences in their application in Spain on a regional level make it difficult to make a comparison amongst Autonomous Communities. It is possible to affirm that even in studies indicating negative effects on the English curriculum, there is significant improvement after the program has been in place for several years. This may be due to the fact that, in recent years, the demand for stronger language skills among teachers has been on the rise. In fact, some studies place a special emphasis on the importance of adequate teacher training and the use of favorable teaching techniques to ensure a successful program [39].

Other authors [40] focus on the evaluation of outcomes achieved in English, concluding that most students perform well in class and achieve excellent levels in both spoken and written English. In addition, they add that written expression in Spanish tends to improve among students participating in the bilingual project. Favorable academic results in English through the bilingual system are supported by numerous studies [41].

The empirical evidence in Europe based on the CLIL model also reveals mixed results, due in part to its heterogeneous implantation and articulation in Europe [1]. A first round of studies [42, 43, 44, 45] reveals positive results in Germany, Finland, Switzerland, Cyprus and Belgium, not only in the learning of native and foreign languages, but in all other subjects as well. By contrast, other authors [46] have found no significant differences in the learning of one’s mother tongue or other subjects, although this lack of difference has been interpreted negatively, given the larger costs that come with a bilingual education. These research projects have come out of Holland, Finland and Switzerland, respectively.

In addition to factors related to individuals and schools, empirical studies generally include institutional variables including: the influence on school performance of whether a school is public or private (whether entirely private or charter). Here, the conclusions are uneven. Some research shows [47] positive effects in the case of private or charter schools, while others [21] insist that this distinction has no effect at all [20]. It has even been claimed that studying in charter schools has a negative influence, while studying in a public school has no influence at all [15]. The controversy is ongoing [48] and new evidence has been found in favor of all subjects in private schools in the region of Madrid, even after controlling for variables as significant as the number of immigrants [11, 26]. Another institutional factor considered influential in recent research is the introduction by educational institutions of standardized tests. It is thought that this has a positive effect given the impact exerted on teacher behavior and the Administration itself [33, 49].

A review of empirical literature does not present clear conclusions as to the impact on academic performance of variables generally studied related to the school, individuals and institutions. However, the majority of these studies concur in the identification of principle determining factors, with an extremely high relevance. These are representative variables from the student’s socio-economic environment, such as the parents’ educational level and occupational category, and various representative parameters as to the cultural customs in the home not only of the student himself, but of his classmates [11, 13, 21, 24, 25, 32, 49].

Bilingual learning in particular seems to have positive or negative effects depending on which study is consulted. There is a need for ongoing research into the academic results coming out of the Bilingual Program in the Region of Madrid, above all because its implementation is recent, and certain studies suggest an improvement in performance over time. The present paper aims to enrich the knowledge and debate on existing materials in Spain. To this end, our model includes practically all of the variables related to individual/schools, institutions and socio-economics that have been taken into consideration in previous studies, including those to exam academic failure, and extreme relevance is given to whether or not a school is bilingual in nature.

## Data and methods

The target of this study is to determine the role played by bilingual education in the academic performance for Mathematics, Science and Technology, Spanish Language Arts and English Language Arts. To this end, two different models will be analyzed. The first of these is a regression model to identify the explanatory variables that influence outcomes (the significant variables from this model are then used in the second model). The second model is a multinomial logit model for determining the variables that influence the probability of obtaining better results. In this case, the dependent variable is divided into four grades related to the Final Evaluation Test given to sixth grade students from the Region of Madrid in the academic year 2016–2017.

This standardized test was introduced in 2015 through the approval in Spain of the Organic Law on the Improvement of the Quality of Education, replacing the previous Test for Essential Knowledge and Skills. It is compulsory for all sixth grade students in the Region of Madrid regardless of who runs the school and whether or not it is bilingual. Like the Organization for Economic Cooperation and Development’s PISA test, the Final Evaluation Test has no academic consequences for students.

In 2017, a sample of 1,667 educational institutions was used from the Education and Research Department for the Region of Madrid. Table 1 shows the sample of schools and students used in this study.

**Table 1:** Sample of educational institutions and students.

	Educational institutions			Students		
	Bilingual	Non-Bilingual	Total	Bilingual	Non-Bilingual	Total
**Public**	286	468	754	22626	12547	35173
**Private**	105	407	512	8575	21363	29938
**Charter**	97	304	401	8110	15689	23799
**Total**	488	1179	1667	39311	49599	88910

### Variables used

The dependent variable is the academic results, which are divided into four grades according to the mark obtained for each school. These grades are shown in **Table 2**.

**Table 2:** Grades of Mathematics, Science and Technology, Spanish Language and English Language scores.

Grades	Score band	Mathematics	Science &Technology	Spanish language	English language
1: D	[0.0–4.9]	[0–319]	[0–332]	[0–355]	[0–331]
2: C	[5.0–6.9]	[320–478]	[333–477]	[355–500]	[322–479]
3: B	[7.0–8.9]	[479–637]	[478–622]	[501–645]	[480–627]
4: A	[9.0–10.0]	> 638	> 623	> 646	> 628

The model will include many explanatory variables that have been previously utilized in earlier studies (for example, immigration, percentage of students repeating a grade, material and human resources, etc.). In addition, we have used other lesser-used variables (which might be considered a contribution to the present research), including the following: school absenteeism, the satisfaction index of families and students, students with compensatory education, whether exams are corrected internally or externally, and the different modes of evaluation used by teachers.

The explanatory variables can be arranged in the three following groups: the individual and the educational institution (school), socio-economic factors and institutional variables.

Our first contribution is to include *bilingual education* as an educational institution (school) to analyze the impact on academic performance in the Region of Madrid.

The available data is for the level of the center. And the variables included in each group are as follows:

1. Variables related to the individual and the educational institution:
  - BILING: Represents the bilingual character of the educational institution in sixth grade.
  - DAT: Area in which the educational institution is located within the Region of Madrid (North, East, Capital city, West and South).
  - SCHSIZE_1: The size of the school is represented by the total number of sixth grade students in the educational institution.
  - SCHSIZE_2: The size of the school is represented by the total number of sixth grade groups.
  - REPEAT: Percentage of sixth grade students who have repeated a year.
  - PRE_PRIMARY <2: Percentage of sixth grade students with early childhood education: before their second birthday.
  - PRE_PRIMARY >2: Percentage of sixth grade students with early childhood education: after their second birthday.
  - IMMIG_1: Percentage of sixth grade students with at least one parent who is an immigrant.
  - IMMIG_0: Percentage of sixth grade students neither of whose parents are immigrants.
  - ABSENT: Percentage of sixth grade students who are absent from class once or twice a week.
  - FAMSTUDSAT: Level of satisfaction with the educational institution among sixth grade students and their families.
  - STUDSAT: Level of satisfaction among sixth grade students with their teachers.
  - TEACHSAT: Level of satisfaction among sixth grade teachers with their profession.
  - SPECIAL: Percentage of sixth grade students with special needs. These students might have a learning delay or problems with classroom integration, among other challenges.
  - COMPENS: Percentage of sixth grade students receiving compensatory education. These students have cognitive impairment.
  - PERSMAT: Level of availability of human and material resources in sixth grade classes.
  - STAB: Percentage of job stability among sixth grade teachers.
  - CULTEC_0: Percentage of sixth grade students who never or almost never use cultural and technological resources.
  - CULTEC_1: Percentage of sixth grade students who use cultural and technological resources once or twice a week.
  - EVA_A: Percentage of sixth grade teachers who use a self-created exam as an evaluation method.
  - EVA_B: Percentage of sixth grade teachers who have students that answer questions individually in front of the class as an evaluation method.
  - EVA_C: Percentage of sixth grade teachers who make written observations about their students’ work and give them a grade as an evaluation method.
  - EVA_D: Percentage of sixth grade teachers who use student self-assessments as an evaluation method.
2. Socio-economic factors:
  - BOOKS <100: Percentage of sixth grade students with fewer than 100 books in the home.
  - BOOKS >200: Percentage of sixth grade students with more than 200 books in the home.
  - EDUC_1: Percentage of sixth grade students whose parents did not complete compulsory education.
  - EDUC_2: Percentage of sixth grade students whose parents have a secondary or post-secondary education.
  - OCUP_0: Percentage of sixth grade students whose parents are unskilled employees.
  - OCUP_1: Percentage of sixth grade students whose parents are skilled/qualified employees.
3. Institutional variables:
  - SCHLTYPE: Ownership of the school: state (PUBLIC), charter (publicly subsidized private school (PRIVPUB) and private (PRIVATE).
  - INTEST: Mode of correction for the sixth grade Final Evaluation Test. i.e. by personnel inside the educational institution. If correction is carried out by teachers outside the educational institution, the variable is identified as EXTEST.

### Specification of the variables that have influenced performance in Mathematics, Science and Technology, Spanish Language Arts and English Language Arts

A robust model using the generalized least squares method corrected for heteroscedasticity has been used (there are no autocorrelation problems, since we are working with cross-section data) to determine the significant variables that explain the scores obtained by sixth grade students in Spanish Language Arts, Mathematics, English Language Arts, and Science and Technology.

In order to analyze some of the characteristics that may influence grades, different dummy variables have been created in the various categories corresponding to the ownership of the center (public, private or charter) and to the location (north, east, capital city, west and south) as well as the various relationships among these.

However, to avoid problems of multicollinearity, in the estimation of the different models the constant has not been included, nor have all the categories corresponding to each variable. For example, with regard to the ownership of the center, private centers serve as the reference variable. In the interrelation between location, ownership and bilingualism in the final model, none of the variables with an estimated coefficient of zero have been included. The results of the estimated model are shown in Table 3.

**Table 3:** Estimation of a regression model using the generalized least squares method to determine which variables have most influenced scores in Mathematics, Science and Technology, Spanish Language Arts and English Language Arts (2016–2017 academic year).

	Mathematics		Science and Technology		Spanish Language		English Language	
INDIVIUAL AND SCHOOL VARIABLES	Coefficient	t-prob	Coefficient	t-prob	Coefficient	t-prob	Coefficient	t-prob
BILING	-0.054	0.140	0.025	0.445	0.037	0.301	0.070	0.058
DAT_NORTH	6.187	0.102	6.209	0.101	6.200	0.201	6.215	0.110
DAT_EAST	6.198	0.101	6.219	0.110	6.192	0.200	6.196	0.109
DAT_CAPITALCITY	6.183	0.100	6.218	0.103	6.186	0.112	6.196	0.100
DAT_WEST	6.194	0.110	6.207	0.098	6.190	0.104	6.227	0.390
DAT_SOUTH	6.198	0.096	6.224	0.105	6.178	0.120	6.212	0.210
REPEAT	-0.159	0.000	-0.109	0.000	-0.164	0.000	-0.157	0.000
PRE_PRIMARY<2	-0.084	0.130	-0.007	0.863	-0.022	0.591	-0.095	0.202
PRE_PRIMARY>2	-0.091	0.120	-0.008	0.802	-0.020	0.600	-0.071	0.269
IMMIG_1	-0.052	0.050	-0.073	0.020	-0.014	0.047	-0.042	0.021
IMMIG_0	0.01	0.620	0.013	0.491	0.012	0.557	0.045	0.307
ABSENT	-0.105	0.030	-0.165	0.000	-0.227	0.000	-0.251	0.000
FAMSTUDSAT	0.008	0.000	0.009	0.000	0.006	0.006	0.010	0.000
STUDSAT	0.004	0.000	0.006	0.005	0.018	0.000	0.006	0.005
TEACHSAT	-0.013	0.270	0.001	0.927	0.001	0.894	-0.018	0.118
SPECIAL	-0.051	0.010	-0.088	0.016	-0.114	0.044	-0.150	0.031
COMPENS	0.063	0.020	0.072	0.046	0.102	0.010	0.119	0.004
SCHSIZE_1	-0.001	0.180	0.000	0.151	0.000	0.716	-0.001	0.125
SCHSIZE_2	0.019	0.370	0.011	0.238	0.000	0.991	0.015	0.125
PERSMAT	0.002	0.140	-0.001	0.121	0.001	0.137	0.001	0.247
STAB	0.019	0.120	0.008	0.452	0.020	0.094	0.009	0.474
CULTEC_0	0.003	0.910	0.004	0.841	0.039	0.242	0.000	0.980
CULTEC_1	0.073	0.030	0.081	0.009	0.019	0.148	0.042	0.001
EVA_A	0.03	0.260	0.006	0.619	0.022	0.178	0.034	0.121
EVA_B	0.002	0.880	-0.003	0.785	-0.014	0.243	-0.001	0.938
EVA_C	0.026	0.270	0.023	0.154	0.007	0.600	0.006	0.670
EVA_D	-0.006	0.530	0.003	0.675	-0.004	0.633	0.000	0.967
**SOCIOECONOMIC FACTORS:**								
BOOKS <100	-0.033	0.220	-0.025	0.307	-0.032	0.231	-0.049	0.068
BOOKS >200	0.014	0.660	0.039	0.168	-0.002	0.943	0.024	0.449
EDUC_1	0.001	0.980	-0.110	0.629	0.030	0.485	-0.015	0.728
EDUC_2	0.066	0.010	0.076	0.055	0.033	0.008	0.039	0.006
OCUP_0	-0.03	0.390	-0.010	0.759	-0.045	0.181	-0.100	0.104
OCUP_1	0.031	0.030	0.038	0.010	0.027	0.006	0.075	0.003
**INSTITUTIONAL VARIABLES:**								
PUBLIC	-0.034	0.110	-0.017	0.133	-0.030	0.114	-0.081	0.120
PRIVPUB	-0.01	0.410	-0.002	0.834	-0.023	0.082	-0.030	0.112
INTEST	0.012	0.170	0.006	0.442	0.010	0.247	0.007	0.408
BILING PUBLIC	0.05	0.230	-0.055	0.133	-0.054	0.176	0.021	0.616
NORTH BILING PUBLIC	0.004	0.900	0.012	0.629	0.013	0.615	0.033	0.217
EAST BILING PUBLIC	0.019	0.440	-0.001	0.956	0.029	0.219	0.065	0.007
CAPITAL BILING PUBLIC	0.035	0.100	0.007	0.709	0.038	0.065	0.065	0.102
SOUTH BILING PUBLIC	0.004	0.840	0.017	0.392	0.031	0.145	0.056	0.091
BILING PRIVPUB	0.009	0.860	-0.035	0.416	-0.016	0.741	-0.045	0.350
NORTH BILING PRIVPUB	0.023	0.660	-0.027	0.555	-0.044	0.372	0.031	0.538
EAST BILING PRIVPUB	-0.171	0.130	-0.298	0.093	-0.063	0.560	-0.066	0.554
CAPITAL BILING PRIVPUB	0.046	0.170	-0.016	0.599	-0.030	0.348	0.030	0.362
SOUTH BILING PRIVPUBLIC	0.053	0.200	-0.028	0.436	-0.001	0.977	0.028	0.487
R^2	0.528748		0.666146		0.528748		0.580949	
Normality test:	Chi^2(2) = 262.6[0]**		Chi^2(2) = 277.6[0]**		Chi^2(2) = 262.6[0]**		Chi^2(2) = 543.8[0]**	
Hetero test:	F(74.1139) = 0.93[0.6]		F(74.1139) = 0.91[0.6]		F(74.1139) = 0.9[0.6]		F(74.1139) = 1.1[0.3]	
RESET test:	F(1.1213) = 0.65[0.4]		F(1.1213) = 0.013[0.9]		F(1.1213) = 0.65 [0.4]		F(1.1213) = 2.78 [0.1]	

p-value is offered in brackets.

** Null hypothesis is rejected for a significance level of 5%.

At a significance level of 5%, the statistically significant variables in different estimated models are as follows:

1. In the case of variables involving individuals or educational institutions: the percentage of students repeating a grade, the percentage of students whose parents are immigrants, absenteeism, student satisfaction levels, satisfaction levels among families and students, the percentage of students with special needs, the percentage of students receiving compensatory education, and the percentage of students who use cultural and technological resources once or twice a week.
2. In the case of institutional variables: the percentage of students whose parents have a secondary or post-secondary education and the percentage of students whose parents are qualified employees.
3. There are no statistically significant variables in the case of institutional factors.

The explanatory capacity of the various models has exceeded 50% in all cases, which is considered to be acceptable for an analysis of this type.

### Descriptive statistics

The statistically significant variables obtained will be used to estimate the multinomial logit model. However, after estimating this model it is important to know whether there are any differences between bilingual and non-bilingual educational institutions for these variables. To that end, some descriptive statistics will be calculated. Table 4 shows these descriptive statistics for both types of schools.

**Table 4:** Descriptive statistics of explanatory variables for bilingual and non-bilingual educational institutions.

	Mean		Standard Error		PearsonVariation Coefficient	
	Bilingual	Non-Bilingual	Bilingual	Non- Bilingual	Bilingual	Non- Bilingual
REPEAT	0.096	**0.150**	0.097	0.148	1.01	**0.99**
IMMIG_1	**0.092**	0.086	0.074	0.092	**0.80**	1.07
EDUC_2	**0.650**	0.533	0.225	0.278	**0.35**	0.52
OCUP_1	**0.503**	0.406	0.248	0.286	**0.49**	0.70
ABSENT	0.032	**0.056**	0.045	0.084	**1.38**	1.51
FAMSTUDSAT	**9.133**	8.864	2.802	3.448	**0.31**	3.89
STUDSAT	**9.239**	9.205	2.716	3.005	**0.29**	0.33
SPECIAL	0.035	**0.045**	0.036	0.058	**1.01**	1.29
COMPENS	0.040	**0.067**	0.070	0.103	1.76	**1.54**
CULTEC_1	**0.455**	0.409	0.140	0.181	**0.31**	0.44

Using the obtained results, it is possible to point out the following aspects related to the significance of these variables in bilingual centers:

- The descriptive statistics point to differences between bilingual and non- bilingual schools.
- The mean found in most variables is higher in bilingual centers than non- bilingual centers. For example, it is possible to highlight the percentage of students whose parents are not immigrants; the percentage of students whose parents have a higher level of education; the percentage of students whose parents are qualified employees; and the higher the satisfaction level among parents, students and teachers, the higher the mean for bilingual centers. This final dynamic also indicates a greater volume of resources.
- The Pearson coefficient of variation shows that most variables have a higher representative mean in bilingual centers than in non-bilingual centers.

This provides us with the basis for selecting the most relevant variables in bilingual centers and also indicates how the variables that are more important in bilingual centers influence the probability of improved test results.

### Analysis using multinomial logit models

After transforming the results into variables (see Table 1), to determine the probability of choosing a specific level based on a set of values that consider the explanatory variables on which this depends, it is appropriate to use multinomial logit models. These models are characterized by discrete choice or qualitative response, and the dependent variable includes a number of integer values [50].

Multinomial logit multiple choice models to determine the probability of choice in each of the different grades can be generated by random utility models, so the utility of choosing the *j-th* grade for the *i-th* center will be given by the following expression:

$$U_{ij}=\beta `x_{ij}+\varepsilon_{ij}$$ (1)

Thus, a center can determine the utility provided by the various alternatives, so that if it chooses one in particular (for example. the *j-th*), and acts in a rational manner, it will be because that alternative is the one that provides maximum utility. i.e.,

$$\mathrm{Pr}ob(U_{ij}>U_{ik})\;\forall k\neq j$$ (2)

If the chosen alternative is represented by *Y*_*i*_ and the *j*-perturbations are independent and identically distributed with log-Weibull or extreme value distribution, which is an exponential function despite its logarithmic transformation allowing for its use as a linear function [51], this function is given by the expression,

$$F\left(\varepsilon_{ij}\right)=\exp(-e^{-\varepsilon_{ij}})$$ (3)

in this way,

$$P\left(Y_{i}=j\right)=\frac{e^{\beta `x_{ij}}}{\sum_{j=1}^{J}e^{\beta `x_{ij}}}$$ (4)

where the independent variables of the model are represented by *x_ij_*.

These exogenous variables can be quantitative or qualitative.

The probabilities of the *J+1* alternatives can be calculated from the estimated equations. It is important to note that in order to eliminate the indeterminacy presented by the model, the PcGive module from Oxmetrics [52] takes β_0_ = 0, so the first alternative will be referenced, in our case the results of those with an insufficient score on standardized tests.

To obtain the various probabilities, the following expressions are used:

$$\begin{array}{l} P\left(Y=j\right)=\frac{e^{\beta_{j}^{`}x_{i}}}{1+\sum_{k=1}^{J}e^{\beta_{k}^{`}x_{i}}}\mathrm{para}\;j=1,2,\cdots ,J\\ P\left(Y=0\right)=\frac{1}{1+\sum_{k=1}^{J}e^{\beta_{k}^{`}x_{i}}} \end{array}$$ (5)

From the values of the sample, maximum likelihood will be used to estimate the values of the coefficients that allow calculation of the maximum probability of obtaining the values of the dependent variable. However, these maximum likelihood estimations are not direct, and iterative methods are used for their calculation. In this study we will make use of the Newton-Raphson method [53].

The expression of the likelihood function for the multinomial logit model is as follows:

$$\ln L=\sum_{i=1}^{n}\sum_{j=0}^{J}d_{ij}\ln P\left(Y_{i}=j\right)$$ (6)

where d_ij_ takes value 1 if *individual i* chooses *alternative j*. and 0 if otherwise.

## Results

Once the model is estimated to explain the probability of improvements in school test scores, where the reference grade is D (insufficient). the results of the estimation of parameters and the value of the Wald statistic are offered (see **Table 5**).

**Table 5:** Results of the estimated multinomial logit model.

	Spanish Language		English Language		Mathematics		Science and Technology	
	Coefficient	Wald	Coefficient	Wald	Coefficient	Wald	Coefficient	Wald
**CONSTANT(C)**	1.702	24.62	-2.900	4.84	1.451	17.46	0.719	4.39
BILING(C)	0.087	0.41	-0.346	0.52	-0.118	0.66	0.676	1.52
REPEAT(C)	-3.573	21.01	-12.967	5.54	-3.321	18.77	-3.250	19.88
IMMIG_1(C)	0.471	0.41	0.337	0.01	0.306	0.16	-0.706	0.79
EDUC_2(C)	1.143	4.09	3.705	2.49	1.272	4.93	0.698	6.99
OCUP_1(C)	1.160	5.04	5.238	5.55	1.280	5.73	1.994	13.78
ABSENT(C)	-1.221	0.77	-24.851	2.97	0.710	0.30	0.865	0.54
FAMSTUDSAT(C)	0.047	1.20	0.443	2.03	0.032	0.51	0.057	1.71
STUDSAT(C)	0.105	5.10	-0.250	0.21	0.121	6.49	-0.024	0.26
SPECIAL(C)	1.896	1.42	7.617	6.26	5.420	11.32	0.431	0.09
COMPENS(C)	-1.469	2.74	2.811	0.77	0.542	0.35	-1.254	2.08
CULTEC_1(C)	0.307	0.40	4.951	8.11	0.030	0.004	0.139	0.07
**CONSTANT(B)**	-6.374	5.85	1.239	9.82	-30.726	1.99	-1.715	3.07
BILING(B)	0.586	0.40	2.841	5.41	-3.944	5.91	-0.358	0.49
REPEAT(B)	-5.765	4.08	-6.135	37.89	-10.635	5.11	-2.795	7.30
IMMIG_1(B)	-2.581	0.27	2.266	5.53	-1.519	0.29	-2.968	0.87
EDUC_2(B)	2.404	5.34	0.564	0.63	6.149	1.32	1.209	0.47
OCUP_1(B)	2.059	4.08	3.311	11.62	5.430	0.74	0.952	0.32
ABSENT(B)	-8.273	4.46	-4.356	5.12	-12.378	4.01	-4.769	4.39
FAMSTUDSAT(B)	-0.163	1.26	0.121	3.93	0.146	0.09	0.585	3.38
STUDSAT(B)	-0.180	1.01	0.039	0.37	1.729	0.68	0.601	3.37
SPECIAL(B)	1.394	0.06	3.160	2.59	22.074	3.19	3.549	2.21
COMPENS(B)	-4.620	1.08	-1.215	1.15	-13.217	1.14	-3.937	0.98
CULTEC_1(B)	0.882	0.11	1.349	4.55	9.443	3.55	3.895	6.32
**CONSTANT(A)**	-0.462	0.12	-2.812	3.01	-2.195	3.35	-1.184	0.006
BILING(A)	-0.620	0.28	-1.764	2.46	-0.881	1.78	-3.636	2.23
REPEAT(A)	0.745	0.01	5.285	2.47	2.403	0.63	7.132	1.85
IMMIG_1(A)	-3.782	0.28	-1.938	0.18	-9.090	4.13	-1.405	26.09
EDUC_2(A)	6.633	2.41	2.316	0.69	2.965	1.72	2.259	0.14
OCUP_1(A)	5.469	1.79	3.610	1.77	3.198	2.35	4.917	0.67
ABSENT(A)	-4.199	0.01	-13.899	8.72	-1.172	0.05	-7.844	1.52
FAMSTUDSAT(A)	0.579	0.99	0.337	6.78	1.206	12.01	0.079	0.05
STUDSAT(A)	-0.584	0.96	0.008	0.003	1.252	11.66	0.346	0.04
SPECIAL(A)	7.790	1.86	6.615	3.46	8.867	10.73	3.676	0.08
COMPENS(A)	-5.090	0.37	0.187	0.002	2.858	0.85	-2.323	0.06
CULTEC_1(A)	3.136	0.85	0.191	0.005	-1.734	1.01	4.272	0.25
**McFadden R**^**2**^	0.5589		0.5609		0.5541		0.5619	

It should be noted that the Wald statistic only serves to provide a comparison for a single parameter and not a comparison amongst various models. It follows a distribution $\chi_{1}^{2}$ and the critical value is 3.84 at the 5% significance level.

Based on the results obtained for the estimated multinomial logit model for the grades of scores in accordance with the outcomes of sixth grade tests in the Region of Madrid, an analysis of the Wald statistic shows that the significant effect of the variables in each of the groups is different. However, the excellent grade is the one that presents the least statistically significant variables independently of the subject. Our model shows that the effect of bilingualism and other variables on the probability of obtaining better outcomes is as follows:

- Bilingualism contributes positively to the probability of improving performance in the subject of English Language Arts. In all other subjects and grades it is not proven to be statistically significant (except in the B- grade of Mathematics where it is statistically significant and negative, which implies that it negatively influences the probability of improving performance).
- When the percentage of students repeating an academic year is statistically significant, regardless of subject and grade, estimated values are negative, which implies that the higher the percentage of repeating students, the lower the probability of improving performance.
- The percentage of students who have a parent who is an immigrant, in general, is not statistically significant in most grades and subjects. However, in the A-grade of Mathematics and Science and Technology, it is significant and negative, which could imply that the probability of obtaining optimal results in these subjects does not improve as the percentage of students with immigrant parents increases.
- The percentage of students with parents who have at least a high school education and the percentage of students with parents who are skilled employees demonstrate very similar behavior, i.e. where statistically significant, they contribute to increasing the probability of improving performance in the C- and B-grades. However, in the A-grade they do not influence the probability of improving or worsening performance in the various subject grades studied.
- With regard to the percentage of students who are absent once or twice a week, it has been observed that the higher this rate, the lower the probability of improving performance. This effect is greater in the grades that include better outcomes.
- Levels of satisfaction among families and students have a very similar behavior in the various grades, such that if it is statistically significant, this implies that the higher the satisfaction level, the greater the probability that the school’s scores will improve in the various subjects.
- The percentage of students with special needs, if statistically significant, positively contributes to the probability of improving performance.
- However, the percentage of students receiving compensatory education is not statistically significant in any of the grades, which implies that it has not influenced the probability of improving school scores.
- The percentage of students who make use of cultural and technological resources once or twice a week, when it is statistically significant, positively influences the probability of improving school performance.

The goodness of fit for the various estimated models, given in this case by McFadden’s R^2^, exceeds 0.55 in all cases. Therefore, it can be seen as quite acceptable for an analysis of this kind. Similar results have been obtained by Nagelkerke’s R^2^ and Cox and Snell’s R^2^, for this reason they have not been included in this analysis.

The classification of each of the groups in order to determine whether or not the model is classified well is offered in the following tables:

The success ratio for the model classification can be calculated, among other methods, by using the success or responsiveness rate of the model (final column of Tables 6, 7, 8 and 9. This rate is calculated by dividing the results obtained through the model by the total number of accurate responses and inaccurate responses). Además, también hemos obtenido the global success rate of the estimated model, (this is calculated by dividing the sum of the diagonal figures of Tables 6, 7, 8 and 9, by the total number of observations). These rates rise to 65.6% in Spanish Language Arts, 81.3% in English, 70.14% in Mathematics and 86.5% in Science and Technology. Considering both measurements, we can affirm that the model is classified quite well in all categories grades of the various subjects since the margin of error is very small.

**Table 6:** Observed and classified values in Spanish Language Arts.

	D	C	B	A	Observed	Success rate
D	340	223	0	0	563	60,39%
C	193	469	3	0	665	70,52%
B	2	5	5	0	12	41,66%
A	1	1	0	4	6	66,67%
Classified	536	698	8	4	1246	

**Table 7:** Observed and classified values in English Language Arts.

	D	C	B	A	Observed	Success rate
D	568	0	104	0	672	84.52%
C	10	12	3	0	25	48.00%
B	111	0	425	1	537	79.14%
A	2	0	1	9	12	75.00%
Classified	691	12	533	10	1246	

**Table 8:** Observed and classified values in Mathematics.

	D	C	B	A	Observed	Success rate
D	524	174	0	0	698	75,07%
C	188	336	0	0	524	64,12%
B	0	1	4	0	5	80,00%
A	4	5	0	10	19	52,63%
Classified	716	516	4	10	1246	

**Table 9:** Observed and classified values in Science and Technology.

	D	C	B	A	Observed	Success rate
D	682	105	0	0	787	86,66%
C	50	382	0	0	432	88,43%
B	5	6	14	0	25	56,00%
A	0	2	0	0	2	0,00%
Classified	737	495	14	0	1246	

Finally, since the model is classified adequately, some examples are given below to determine the probability of each of the four levels corresponding to the various results. Thus, for example, if we use the average sample values of each of the variables that encompass the characteristics of the schools, the socio-economic environment and the students; the estimated values of the probabilities of the four levels of results established for the schools would be those that appear in Table 10 for each of the subjects.

**Table 10:** Estimated probabilities.

	Probabilities Spanish Language	Probabilities English Language	Probabilities Mathematics	ProbabilitiesScience and Technology
D	0.44982	0.60424	0.54173	0.57734
C	0.54793	0.0021692	0.017657	0.41659
B	0.0017603	0.39009	0.43098	0.0000031
A	0.00048672	0.0034991	0.0096308	0.0060669

According to these results, it is highly probable that most schools have the highest percentage of students in the D grade in the subjects of English Language Arts, Mathematics and Science and Technology, regardless of the ownership of the school and whether or not it is bilingual. However, in Spanish Language Arts the C grade is more likely.

On the other hand, although it is not the most probable, it must be noted that in Mathematics and English Language Arts, there is a high probability that a considerable percentage of students will attain the B- grade.

Finally, the probability that schools will have the highest percentage of their students in the A- grade is practically nil in all subjects.

## Discussion

In recent years, an impressive commitment has been made to bilingual learning in Spain, especially in the Region of Madrid. This is associated with rising costs in public education without conclusive empirical studies that have provided results as yet on the efficacy of such a project. The effects of bilingual learning on academic performance can be considered positive or negative, depending on which studies are consulted. It is therefore necessary to continue to study the efficacy of the Bilingual Program in the Region of Madrid, given that its implementation is quite recent, and certain studies indicate a greater impact over time. The present paper aims to enrich the current knowledge and conversation on the subject, in an effort to delve deeper into what is known surrounding the effects of a bilingual education on academic performance in the various subjects taught in primary school.

In the first phase of the study, a generalized least squares method has been used, including practically all individual/school, institutional and socio-economic variables considered in previous studies, with special attention paid to whether or not the school engages in bilingual learning. This first phase serves to identify the most significant independent variables, according to the results shown in Table 3. In the second phase of the study, a multinomial logit model has been used in order to analyze the factors that influence the probability of obtaining better academic outcomes using the variables that were statistically significant (once these have been categorized), with special attention paid to bilingualism. These results have been offered in Table 5.

Based on these findings, it has been concluded that the CLIL program in the Region of Madrid does not influence academic performance in those subjects taught in English, namely Science and Technology. Neither are subjects taught in Spanish affected: Spanish Language Arts and Mathematics. It is not relevant whether or not the center is public, private or a charter school. In the subject of English Language Arts, an overwhelmingly positive tendency has been shown as to the probability of achieving better academic outcome [54].

Our results are in line with certain studies conducted previously [1], with the analysis extending to three CLIL programs in different single-language Spanish regions (Andalusia, Extremadura and the Canary Islands). Conclusions in a study of primary and secondary schools do not indicate a decline in school performance when it comes to subjects taught in English, namely Natural Science, and neither are skills in Spanish Language Arts and English Language Arts affected.

By contrast our results obtained from this study differ markedly from other assessments carried out in various regions of Spain. These attributed a negative effect to bilingual education in the performance of subjects taught in English [33, 35, 36] although, most especially in the case of the Autonomous Community of Madrid, improvement was observed over time and in more recent years. In any event, it is possible to detect how more recent studies carried out in Spain are showing results that are more similar to those carried out on a European level, where most conclusive research points to the positive effects of bilingual CLIL programs when it comes to subjects taught in both native and foreign languages, and even in the acquisition of knowledge in other subjects [42, 43, 44, 45]. What is certain is that there are also European studies that do not identify any effects at all [46], but most research attributes positive effects to bilingual learning.

Finally, we wish to point out that the positive effects of bilingual education are more affirmed by such projects as the immersion program in Canada and the sorts of bilingual learning programs found in North America (which precede the CLIL program) than they are by the European CLIL program itself. Accordingly, most specialized literature shares the view that there is little effect in the program on the learning of one’s mother tongue and on the outcomes for subjects taught in the foreign language, although there is an improvement in performance when it comes to foreign language learning [5, 55].

We must stress that immersion programs are different from the European CLIL model, due mainly to the socio-linguistic and socio-cultural differences in which the programs are implemented. But the vast experience associated with immersion programs makes it essential that we pay attention to the results of their findings.

With respect to the other variables included in our multinomial logit model (see Table 5), our results are generally coherent with those obtained in previous studies. Variables that have a negative influence on the probability of obtaining good academic outcomes are: the percentage of students whose parents do not have a secondary education [19, 33], the percentage of students who repeat an academic year [16,32], and school absenteeism [19]. Variables with a positive influence are: parents with a higher level of education [20,21,47], the level of satisfaction among families and students with the good relationship between students and teachers [26], and the technological and cultural resources available in schools and homes [31,32].

The variable representing the percentage of students with at least one parent who is an immigrant is not statistically significant in most grades and subjects except Mathematics and Science and Technology. This shows a negative influence on the probability of obtaining the best possible academic outcomes as [25] have concluded. In this regard, [20] identify a significant negative impact of the condition of immigrant, especially first generation -that is, born abroad and with foreign parents- which becomes much more evident when the proportion of immigrants in the center is high (over 20% according to [21]).

The bilingual program is a significant effort on the part of the Region of Madrid in every sense, not merely in economic terms [4]. Still in the expansive phase, the program has not been fully rolled out, and has still not reached all of the schools in the Region of Madrid. It is important to remain watchful while remaining aware of the cost-benefit ratio.

The results of our research are essential when considering whether or not to continue with an expansionist policy. It is important to note that the main objective here is to improve foreign language skills. One of our determinations is that the bilingual program is indeed effective in pursuit of said goal. This result is especially relevant if we take into account that many of the other variables we considered as determining factors in educational achievement, with the exception of parents’ educational level and the number of books owned by the family, tend to have a negative effect, as indicated above. The improvement of English language skills is a worthy endeavor, especially in Spain and given the prevailing economic and social context of the day.

Nevertheless, it must be pointed out that the present paper has some limitations owing to a lack of available data in certain institutions and areas of the country. This explains why we were generally unable, as part of this study, to contrast our findings with those found in other Regions in Spain during the research period. Another limitation of this study is that the effects of the bilingual program on secondary or tertiary education have not been considered in the Region of Madrid, because their respective data are not available during a consistent period. So, future research should incorporate this type of analysis if data is available.

## Supporting information

S1 Data (XLSX)
